# Retention and Completion of a Doctoral Nursing Programme: Sense Making Through Collective Reflection

**DOI:** 10.1111/jan.70291

**Published:** 2025-10-18

**Authors:** Sally Bassett, Helen Ayres, Karen Lascelles, Elaine Strachan‐Hall

**Affiliations:** ^1^ Oxford Brookes University Oxford UK; ^2^ Oxford Health NHS Foundation Trust Oxford UK; ^3^ North West Ambulance Service Clinical Associate KPMG Banbury UK

**Keywords:** academic development, attrition, community of practice, doctorate, nursing, reflection, sisterhood, support

## Abstract

**Introduction:**

This discussion paper explores the group experience of a cohort of eight nurses completing our university's first professional nursing doctorate programme.

**Aims:**

This paper aims to make sense of our shared experience and to contribute to what is known about doctoral study by sharing our insights.

**Design:**

Discursive paper.

**Methods:**

Through individual and group reflections on our experience, we address the questions ‘why did we stay’? and ‘how do we make sense of the fact that we all, as a group, successfully completed the programme’? We drew on principles of collaborative and collective auto‐ethnography to guide our group reflexivity in response to these questions.

**Findings:**

The main reasons we gave for staying were: (i) commitment, which had three strands ‐ ‘proving’, ‘obligation’ and ‘self‐determination’ ‐ and (ii) shared‐identity and common humanity. The two further elements that helped us make sense of our cohort's completion were (i) the joy of learning together and (ii) professional friendship and Socratic inquiry.

**Conclusion:**

As the first programme cohort for the nursing doctorate in our area, we became a close and supportive group, which we argue contributed to our success. We ascribed this to our characteristics as doctoral students and the creation of a sisterhood reminiscent of a community of practice. We also acknowledged the importance of the WhatsApp platform in facilitating group cohesion, and the sense of reflexive closure brought by the process of reflection at the end of our programme.

**Implications for Doctoral Education in Nursing:**

We recommend that doctoral cohorts, supervisors, and teaching teams systematically plan opportunities into programmes for organic relationship development and consider how the literature on communities of practice and academic persistence might support academic development. Academic staff could also encourage students to set up an online communication channel such as WhatsApp or similar at an early stage in their programmes and give particular consideration to closure and transition to post‐doctoral practice on completion of professional doctorates.

## 1 Introduction

A professional doctorate is a research study programme for those wishing to undertake research that is both based in and contributes to their professional practice. Originating in North America in the 1930s (Ketefian et al. 2005), the professional doctorate was first introduced to the UK in the early 1990s (Bourner et al. 2010). The first programmes were predominantly in the fields of education but have since expanded to virtually all disciplines, including nursing (Rees et al. 2019). The professional doctorate pathway is typically undertaken by those with significant professional experience, and the development of more advanced nursing roles has contributed to an increased demand (Rees et al. 2019). Given the context in which this form of doctoral study is undertaken, students tend to study part‐time alongside their professional roles. Attrition rates for professional doctorates appear to be higher than for the traditional PhD route, and this is likely attributable to the later career stage in which they are undertaken, the competing demands of professional roles, and particularly pertinent to nursing—a predominantly female profession—the demands of family life (Aitchison and Mowbray 2013; Rees et al. 2019). This discussion paper seeks to develop what is known about the experiences of nurses undertaking professional doctorate programmes, focussing on factors that influence successful completion.

## 2 Background

We are a cohort of eight female nurses who, in 2024, completed a nursing doctoral programme in the UK. The programme comprised eight taught modules delivered over seven semesters. The modules were designed to incrementally facilitate students' intellectual, academic and reflexive growth from our various baselines through to doctorate level, leading to the completion of a formal and ethically approved research study conducted during years three to five. Our learning journey was interrupted by COVID‐19, extending the programme into a sixth year. The completion of our theses reflected the culmination of the doctoral programme.

As an inaugural doctoral cohort at our university of study, we comprised registered nurses aged late forties to mid‐sixties on completion. We were a group of experienced mid and senior‐career nurses from diverse settings and specialities within the fields of mental health, adult acute, community, management and policy and education.

The doctoral student's experience can feel like a lone endeavour (Morris et al. 2021), and our professional doctoral programme had been designed to foster a cohort experience through the taught stage, and form a community of practice (Wenger‐Trayner and Wenger‐Trayner 2015) that may extend into the research phase and beyond. Additionally, at the outset, we formed a WhatsApp group to stay in touch between face‐to‐face teaching days. Some of us were familiar with this platform; for others, it was a new phenomenon. The use of the WhatsApp platform was seen as instrumental to our group's development, bonding and ongoing cohesiveness (Bassett et al. 2019).

On completion of our programme, we engaged in rich and enlightening informal reflexive conversations about why we had all completed the programme when attrition for doctoral programmes is known to be a common issue (Cohen 2011; Devos et al. 2017). We were curious as to what it was about our cohort that contributed to us all having successfully reached the end, particularly as we were aware attrition had been higher in subsequent cohorts. We wondered about potential influencing factors; the programme design or characteristics of us as individuals or as a group. We were struck by the words we had used during our informal conversations that described the sentiments and emotions we had experienced, and how this gave meaning to our collective experience of why we stayed. We felt compelled as a group to advance our informal discussions and create a meaningful and collective narrative to explain what made us all stay. Reflectively re‐examining our experience in a more organised way has given further depth to the connectedness we experienced and brought a satisfying closure to the doctoral student journey. The aim of this paper is to share the ways in which we made sense of our experience and to contribute to what is known about doctoral study by sharing our insights with the wider nursing community.

## 3 Method/Approach

Upon completing the doctoral programme and following our successful graduation, we met to reflect on our experiences in a considered and structured way. It was not our intention to undertake a primary research study, and we did not conduct a literature review at the outset; however, we drew on elements of collaborative and collective autoethnography to give us a feeling of rigour and guide our reflective and discursive approach. Autoethnography involves deep reflexivity to illuminate ‘personal connections to—and investment in—identities, experiences, relationships and/or cultures’ (Adams et al. 2014, 27), which resonated with our desire to make sense of our experiences in the context of ourselves, our group, our profession and nursing academia. Outcomes of autoethnographies do not end with the narrative presented by those involved; rather, their meaning is extended through the interpretations of those reading them (Peterson 2015). This principle struck a chord with us due to our wish to share our experiences to interest and inform other doctoral students and educators. Collaborative autoethnography involves multiple researchers constructing individual experiential narratives related to a research topic before collaborating, either fully or partially, to achieve a shared interpretation (Chang et al. 2016), while collective autoethnography is concerned with co‐constructing narratives of communal experiences, typically involving researchers who have a pre‐existing relationship and using group interviews as the medium for reflection (Karalis Noel et al. 2023). We were influenced by both of these approaches, viewing ourselves as a collective and valuing both our individual and our communal experiences. Our process of reflection followed the stages outlined below.

1. Co‐construction of two key questions to stimulate reflection: (1) *Why did you stay? If you thought about leaving, why didn't you?* (2) *How do you explain/make sense of the fact that we all—as a group—successfully completed the programme?*
2. Construction of individual narratives through written reflections.
3. Reading each other's reflections and noting patterns and insights.
4. Discussing our reflections and insights as a collective. This discussion, which we recorded, helped us develop our understanding and appreciation of our individual and collective experiences. To ensure all group members contributed to the discussion, those who could not be present when we met (three of us) contributed perspectives further to listening to the recording.
5. Using the recording and additional comments to form a meta‐reflection transcript containing the insights as a group.
6. Agreeing as a group the application of relevant concepts and theory to elucidate the group's insights and explanations related to the two key questions. It was at this point we engaged with the wider literature (we did not conduct a literature review at the outset of this work but drew on relevant literature as our insights developed, in keeping with autoethnographic principles (Ellis 2004)).

## 4 Insights

The insights we gained through our individual and collective reflections are grouped under the questions we asked ourselves: ‘why did you stay?*’* and ‘how do you explain that we all successfully completed the programme?*’* Quotes are included below, and the ‘voices’ of each of the eight participants are represented in the discussion below. Where a number of similar quotes or phrases were evident across the group, we have included the most representative ‘voice’ to illustrate our reflections.

### 4.1 Question 1: *Why Did We Stay?*

Our responses to the question of ‘why we stayed’ reflected two aspects of our experience; the individual motivations that we had for starting and completing the doctorate, and the part the collective experience played in the success of our cohort. Through our discussions, we recognised that most of these motives revealed themselves to us as individuals only after we had successfully completed the doctorate. We had not previously discussed these motivations in any meaningful or overt way with each other or others; they were private, unspoken and maybe subconscious. What became clear was that, to a lesser or greater extent, despite our powerful individual motivations, we had all rehearsed the idea of leaving the doctoral programme at some stage. Hence, the question ‘why did you stay?’ felt all the more important. The main reasons we gave for staying were: (i) commitment, which had three strands of ‘proving’, ‘obligation’ and ‘self‐determination’ and (ii) ‘shared‐identity’ and ‘common humanity’. These insights are presented and discussed below.

#### 4.1.1 Commitment

Our individual reasons for staying on the programme spoke to the internal factors that contributed to our sense of ‘commitment’. We all recognised that the challenges we encountered as individuals during the programme had led us to either entertain the idea of, or seriously consider, leaving the programme. We all, to some extent, mentally rehearsed what leaving might look and feel like, and some of us recalled verbalising these thoughts and discussing them with peers, friends, family and tutors. We all acknowledged that our decisions to stay were underpinned by a steadfast commitment to the programme and a resolve to succeed. At times, however, our commitment and resolve were significantly tested.

The challenges we faced as doctoral students centred primarily on stressors we experienced in our personal and professional lives. Consistent with the experience of other female doctoral students (Aitchison and Mowbray 2013; Lee et al. 2024), many of us cited family pressures and demands as having prompted us to consider whether continuing on the course was a viable option and the right thing to do. This was particularly difficult when, due to adverse situations including illness, stress and trauma, the needs of family members increased. In addition to this, the COVID‐19 pandemic significantly impacted our professional lives and had a detrimental effect on our mental and physical well‐being. The demands we faced in practice required us to narrow our focus to work and family. This commitment superseded our doctoral desire and capability during the throes of the pandemic, leading to a delay in our studies, and, for some of us, uncertainty about whether or not we could continue at all.

Our individual and collective reflections identified three prominent internal motivating factors contributing to our ongoing commitment: ‘proving’, ‘obligation’, and ‘self‐determination’.

#### 4.1.2 Proving

Among our reflections and discussions, it was striking that many of us felt an impetus to prove to ourselves and/or others that we had the intellectual ability and staying power to complete the programme. This need to prove was predicated on various life experiences. Our reflections and discussions explicitly and implicitly expressed a desire to resolve a sense of academic inadequacy. Linked to this was the determination not to ‘give up’ and to see something through to its conclusion where this may not have previously happened. Whilst our collective reflection concluded that this aspect of our experience was best captured by the term ‘proving’, it resonates also with the concept of ‘grit’. Defined as ‘perseverance and passion for long‐term goals’ (Duckworth et al. 2007, 1087), the presence of grit sees individuals maintain their effort—potentially over years—in spite of challenges and failures.

I wanted to prove to myself that I could complete something. I think of myself as someone who never quite finishes what I set out to do, and I wanted this to be different. I wanted to prove to other people that I was not someone who gives up—I didn't necessarily think people would say that, but I believed it was a perception that some may have of me nevertheless.

Several of us wrote and talked about a fear of failure and feared being ‘the one that failed’ or ‘an attrition number’. For some of us, obtaining a doctorate was a longstanding ambition and a career goal. It was important to prove to ourselves that we could realise this ambition and prove to fellow nurses—and other disciplines—that being a nurse with a doctorate was an achievable and meaningful goal.

#### 4.1.3 Obligation

Our sense of obligation to all those investing in, supporting and enabling us was a significant motivating factor in completing the doctorate. As we progressed through the programme, this sense of obligation intensified. From the outset, those of us who had been awarded funding felt a strong commitment to our funding organisations, whose faith and investment enabled us to embark on the programme. Those of us who self‐funded wanted to ensure family financial sacrifice was not wasted. Similarly, we recognised early on the dedication and commitment of the teaching team—they had invested much effort into developing the programme and guiding us as the first cohort. We felt a deep sense of responsibility and gratitude towards them.

Our obligation to the nursing profession to complete the doctoral programme was articulated in its formative stages and revisited as our studies progressed. This obligation strengthened as we became increasingly passionate about the value of nurse‐led research. We saw ourselves as having an important part to play in securing the status of registered nurses as academically able professionals who can positively and constructively influence nursing practice. As we progressed through the programme, our sense of obligation to others grew, strengthening our resolve as it did. The nature of our studies meant that we spent time talking to participants—be they patients, carers, nurses and multi‐professional team members—many of whom had generously and bravely described what had been difficult and often traumatic experiences for them. They had volunteered to participate in our studies in the hope that their contributions would make a difference, and they relied on us to share their stories. Our responsibility to do justice to these individuals and their experiences was keenly felt, and it represented a powerful driving force for us all.

I couldn't let the participants of my study down. They had generously given their time to talk about their traumatic experiences, and they were invested in my research, relying on me to share their stories.

…we each owed it to our participants to make sure their voices were heard.

As time went on, the impact of our endeavours on our families grew, as did the extent to which we needed their support. We did not want to let them down after the sacrifices they had made to support, or tolerate, our studies. Moreover, we did not want to betray their pride in us as we worked towards achieving our goal, and we felt an obligation in response to their willingness to support us. This resonates with work by Lee et al. (2024) which identified the pivotal support of families, universities and workplaces required to succeed.

As our relationships with our supervisors developed, so did our sense of obligation to them. They were invested in our success, and we saw that it meant success for them as supervisors too. Finally, our sense of obligation extended to the group. The relationships and identity we had developed as a group meant we knew that if we left, it would affect the whole group. We did not want to disrupt, let down, or disappoint our fellow students.

There was a feeling of if I stop now it will affect the whole group—a bit like a collective responsibility.

#### 4.1.4 Self‐Determination

Undoubtedly, our felt need to prove ourselves and our sense of obligation fuelled our individual determination. However, we also believe our self‐determination was more than these elements alone and that it played a crucial role in our journey through the doctorate programme. We were intrinsically motivated to learn and develop academically and professionally. We shared a great passion for our profession, and we relished the opportunity to immerse ourselves collectively in all aspects of nursing. We believed in our studies and, through our research, wanted to contribute to both our areas of practice and nursing as a whole.

We reflected on feeling ‘compelled’ to complete the programme, citing pride, the desire for achievement and the desire to be a Doctor of Nursing as key driving forces. Some of us, who regard ourselves as in the later stages of our professional lives, also described being driven by our wish to leave a legacy.

As we reflected individually and together, we recognised that we all identify as determined women who share the belief that once we commit to something, we see it through. As several of us said, the experience tested and challenged us in many ways, such that we all rehearsed the idea of leaving, but doing so was ‘never an option’.

Having this doctoral opportunity was in line with my whole being of lifetime learning. I wouldn't have missed this. Not completing this was never an option even though at different points of the journey it became tough, and I verbalised thoughts of quitting.

The sentiments we expressed about being self‐determined are reflected in the literature on academic persistence (Lew et al. 2020) and the doctoral students' self‐directed learning experiences (Porter et al. 2020). The work of Lew et al. (2020) on academic persistence identified that where there is a strong intertwining of the social and academic integration of a group in the context of self‐directed learning, then students report a more satisfying experience that is associated with completion of an educational programme. In our group, not all students had universally positive academic experiences. However, the strength of the personal motivations, combined with the camaraderie of the group and the Socratic questioning that we engaged in and discuss later in this paper, significantly contributed to the effectiveness of our approach. This meant that the core requirements of successful self‐direction as described by Lew et al. (2020) were met through the collective group support that moderated the less positive academic experiences.

Similarly, Porter et al. (2020) stated that issues associated with doctoral degree completion are complex, however, the self‐directed learning environment can enable students to progress through their studies successfully. Porter et al. (2020) draw attention to the importance of aligning students' perceptions of their goals and expectations with their aspirations and achievements. This dimension of self‐directed learning resonates with the sentiments that we shared through our reflections and have presented throughout this paper. The expectation for us as doctoral students to manage our learning to promote a high level of critical understanding and its application to our professional practice was explicitly stated in the programme handbook; the programme design, as previously referenced, aimed to create a cohort experience to engender social interaction between us as students. Successful completion of the programme by our group might, then, be attributed to the design of the programme, however, the group felt that the shared learning and social interaction experiences far exceeded what might have been hoped for from these pedagogic interventions.

### 4.2 Question 2: *How Do You Explain/Make Sense of the Fact That We All—As a Group—Successfully Completed the Programme?*

#### 4.2.1 Shared Identity and Common Humanity

From our reflections, it became apparent that a shared sense of group identity was an influential motivating factor for our commitment to the programme. Our shared identity was distinct from our individual characteristics such as our professional roles or grade. We were curious as to the elements that contributed to this shared group identity, such as the initial selection of us as a cohort, the investment from academic staff throughout the programme, group understanding, support and cohesion and our shared passion for nursing.

The doctorate group gave me an identity, my colleagues validated me, it kept me going.

Our student group was distinct in that it was the first cohort. One of our reflections considered that the development of group identity may have started during the selection process through the selection of candidates who were determined and passionate.

I need to credit … the [selection of] appropriate individuals for the first cohort. Although we are very different in many ways and our careers have taken different paths, there are similarities within our personalities that make us all so incredibly determined and passionate.

Similarly, others of us referred to elements of expectation and ambition in the academic staff who had designed and taught this first cohort.

I felt our group was important, not just personally or for our profession, but for [the institution/University] and the lecturers. I felt invested in our identity as ‘forerunners’ and saw us setting out on the programme as the culmination of a huge amount of hard work, ambition and commitment on the part of the teaching team.

The developmental nature of the programme was also described by another of us, who spoke of the academic staff investment in the group:

It was certainly good for the programme as we were able to weather the uncertainties of the embryonic course and help it grow. We were also able to benefit from the tutors' dedication to making it work, and I do think they were hugely invested in us …. as a group.

An important feature of our group identity was a strong desire to be successful and make an impact as a cohort. Others outside the group—such as work colleagues, friends and family—could not experience this sense of collective commitment. This was described vividly by one group member who reflected on the ‘advice from friends and colleagues to give up the programme’ as contrasted with the support from the group as ‘none of us wanted anyone to fail’.

During our reflections on the group identity, the nurturing nature of the group was frequently mentioned. Throughout the doctorate, a consistent distinguishing factor of our face‐to‐face time was eating together. We quickly formed a refreshment modus operandi of who might bring the kettle, mugs, tea, coffee and milk and who would bake or cook. We described this physical nurturing of each other as bonding and nourishing, and our reflections showed that we considered this characteristic to have cemented the collegiate nature of the group. This also overspilled into online conversations; we would eat and drink together online despite being in separate houses. This social aspect of the group activity was perceived as an important strength.

…our sharing of food and drink… that we all did, and that was a very important part of what we did. Because I think sharing food was physical nourishment. I think we nourished each other emotionally, and you know, practically. But I think that was a physical nourishment. It's about connecting, feeding each other, caring, nurturing… It's something we always do when we get together.

Our reflections recorded our belief that using WhatsApp was instrumental in maintaining our group connections and identity. Whilst we did not consider this use of social media to be causative in terms of creating a group identity, it was described as enhancing the connectivity between group members, and creating a medium of support. Initially, much of our online interaction was concerned with sharing practical advice and know‐how. Over time however, we also shared our fears and worries more transparently and responded to each other with advice and support. Our online group was referred to as a safe space, and the instant and shared nature of the platform was considered to have facilitated group awareness which in turn added to the group's shared identity as a caring community.

WhatsApp gave us proximity in the midst of distance and that kept me grounded. I enjoyed the humour, the transgressions, the sharing of lives and family ups and downs, the advice, information sharing and the inherent non‐judgmental ambience that reflected our respectful acceptance in the classroom.

It was a conduit for communication—the medium of our relationship that kept us together. It kept me from feeling alone or on my own—it was my delight. At the beginning, it was a chat group interspersed with ‘how‐to know‐how’. It became a sound‐off opportunity and then a medium of shared experiences. It provided companionship and validation of me.

Our reflections align with the work by Tajfel and Turner (1979) on social identity theory, who posit that individuals derive a portion of their self‐concept from their membership of social groups. They argue that belonging to a group can provide a sense of pride and self‐esteem. Our group reflections suggest that it was not only belonging to a doctoral cohort but being part of the *first* nursing doctorate cohort for our university that was important to us.

Tajfel and Turner (1979) also discuss the need for groups to have a purpose. Some of us expressly referred to a sense of pride and affirmation about our trailblazing status. Our shared goal of doctoral achievement facilitated a quick identification with each other, which crystallised over the course of the programme as we discovered new professional and personal commonalities. While initially we considered, or expected, our intrinsic purpose as a group to share information related to the course requirements, we reflected that this purpose transitioned to the giving and receiving of support specifically related to getting through the course. This resonates with the early work of Durkheim (1897), who is credited with identifying the importance of social relationships in protecting individuals from harm. It might be argued that our shared group purpose grew to prevent the harm of failure. Indeed, many of the participants cited group support, encouragement and validation as key to keeping going. Group social identities have been explored more recently by Haslam et al. (2022) who found that group membership provided individuals with important psychological resources for well‐being. Examples of this were identified from our reflections on the strong sense of emotional support we received from each other, which corresponds with the emotional connectedness cited by Kyprianides et al. (2019) as being key to group identity. For example, one of our reflections described the group's support as a substitute for support at home, because of the implicit understanding of the need to press on to the end, which was not always understood in the same way by those at home. This was endorsed by another of us who commented that the group's support helped her to manage boundaries at home by reducing the burden of support needed from her family. For all of us, it was clear that the ‘knowing’ we shared, of the doctoral stresses, anxieties, demands and expectations, was instrumental in sustaining our drive to complete the programme.

…the other members of the DNurs group … were the only other people that truly understood the demands of academic study whilst working full time in a demanding role … it was the cohort of my fellow students that kept me going.

Our reflections led us to consider further the interconnectedness of our individual and collective experiences. Building on the consensus around identity is the notion of common humanity. This reflects the experience of our cohort, which found that being part of the group was more than merely sharing a nursing registration and a desire to complete a doctoral programme. It was something more emotional and nuanced.

We noticed that competitiveness between us as peers was absent. Instead, we were as invested in each other's successful completion as we were in our own.

We all wanted each other and all of us to do well, to get to the endpoint, and we were all keen to support each other in this shared endeavour.

The group made me feel capable: it was levelling between us.

When I completed the programme, the main emotion I felt was relief. When the whole group finished, I felt joy.

The lack of competitiveness seemed to enhance our sense of safe cohesion: we trusted each other with our study anxieties and our personal and professional stories and challenges. Our reflections conveyed a sense of psychological safety. One described the group as “powerful and safe”, and another as “reassuring and affirming*”*. This was ascribed to the shared endeavour, which in turn, created group solidarity. We described this differently; as the collective goal of everyone's success or a collective responsibility to each other.

We reflected that we had developed a sense of sisterhood; indeed, at some point during the programme, we started to refer to each other as our ‘seven sisters’ (referring to the other seven in the group). This ‘sisterhood’ was almost seductive in facilitating a powerful and sustained connection, and it demonstrated our collective commitment and responsibility towards each other and our group.

I didn't want to be left out of the group. I would have found it really hard knowing the group I valued so much was continuing without me.

I felt a commitment and responsibility towards them.

Our meta‐reflection brought us to a deeper consideration of the sisterhood and the cohesive mechanisms which are around commonality as nurses, women and family members. We reflected on ourselves as family members outside of the group and noted that our cohesion was strengthened by our sharing of familial challenges and celebrations. Our choice of ‘sister’ terminology when referring to ourselves also suggests a sense of family within the cohort. This was inadvertently highlighted by one participant's reflection that had it not been for the doctorate, she may not have been drawn to other personalities within the group, however this did not stymie the development of sisterhood. This could perhaps be equated with the common saying ‘you can't choose your family…’, in this instance having a positive outcome.

Individuals in our group span a variety of roles and responsibilities … I may not ordinarily have been friends with [them] in life had we not come together on the doctoral programme yet I would consider these nurses to be good friends and I am grateful to know them and have them in my life.

As a group of eight women, it is perhaps inevitable that our description of our shared identity is female. However, the familial underpinnings of sisters and the solidarity associated with the term sisterhood helped us explain the way we sustained our commitment to each other throughout, and despite, the challenging terrain of the doctoral journey. Aitchison and Mowbray (2013) highlighted the presence of emotional labour among women doctoral students and observed that they often sought out supportive groups where they could engage in “troubling talk”—a feature of which was being open and honest about their feelings. This was a clear function of our ‘sisterhood’. Being able to express emotions, something frequently rejected by the research culture in higher education (Johnson et al. 2000), reduced emotional dissonance and provided a protective shield against attrition as we could be authentic and labouring together.

Our reflections also accord with various concepts drawn upon to illustrate collegiality and cohesion within nursing. For example, nursing collegiality is defined by Kangasniemi et al. (2024, 597) as ‘achieving mutual goals together, reciprocity, trusted advocacy, powerful self‐regulation and engaged belongingness,’ with the professional connection being an important antecedent. Additionally, the notion of professional generosity, as understood by Horvat (2021) echoes the familial context our reflections revealed. Horvat describes professional generosity as feeling valued professionally through core relationships, which she characterises as underpinned by a sense of cohesiveness and community, family nurturing and unconditional acceptance. We have asked ourselves whether our experience of sisterhood would have been different or existed had the group not been exclusively female, and we sense that it would have been, although there is no way of knowing this. Interestingly, Horvat's research into professional generosity among nurse academics involved only female participants, and this raises the question as to how much of the literature on nurse cohesiveness, collegiality and professional generosity has developed from female experiences, although of course there are also males contemplating related concerns (Thompson and Clark 2018).

#### 4.2.2 Making Sense of Our Cohort Experience of Completion

We asked ourselves how we explained the success of our cohort in contrast to the existing research on attrition rates in doctoral programmes. Two key elements featured in our reflections, first, the joy of learning together and second, our professional friendship and use of Socratic inquiry. These are presented and discussed below.

#### 4.2.3 The Joy of Learning Together About Nursing

As discussed earlier, our reflections highlighted the part physical nourishment played in helping us to form our bond as a group. The words we used in our reflections speak of nourishment in relation to our experience of collective learning about nursing. It was through this shared intellectual experience that we found joy in re‐examining the meaning and value of nursing. It is perhaps naïve to think that we wouldn't have developed our appreciation of nursing, but what was unexpected was the joy we gained from coming together and immersing ourselves in nursing discourse, theory and values.

The taught part of the programme was a real joy for me. I looked forward to Saturdays, and devoured the rich and diverse conversations we had about nursing.

The taught years were like nectar. Saturdays were a nurturing joy, sharing wisdom [and] encouragement and I drew inspiration and a deeper understanding of nursing.

…the pleasure I got from being with expert nurses, talking about nursing.

The collective experience of unravelling the complexity of nursing as an art and science felt enlightening. We gained a shared appreciation of what it meant to be a registered nurse, and to practice nursing. Being able to articulate the essence of nursing between ourselves, in our writing, and to others as we did was immensely satisfying, profound and transformative. This transformative process, where we passed through a threshold in our understanding and appreciation of nursing, was one where our professional knowledge was re‐contextualised to serve a new purpose (Meyer and Land 2005; Van Schalkwyk et al. 2019). This meant our individual and collective knowledge was bound to become new disciplinary knowledge, explained as transformative (Meyer and Land 2005; Van Schalkwyk et al. 2019) but experienced as joyous and the forging of a greater love of nursing. Hence what became clear to many of us, was that this intellectual nourishment reaffirmed our love of, and commitment to, nursing.

The work of Adib‐Hajbaghery et al. (2021) and Bolandian‐Bafghi et al. (2022) found that the love of nursing was significantly influenced by the appreciation of nursing holding a ‘specialist body of knowledge’ and, through academic endeavour, the continuous expansion of this professional knowledge. Possessing a specific body of knowledge was considered to influence the public's perception of nursing positively, and as senior nurses, we were cognisant of this. As our appreciation of nursing knowledge grew, our motivation and commitment to honour the contributions made by our participants and to complete our work grew. We were driven by our awareness that completion of our doctoral research would create new nursing knowledge that could contribute to expanding the profession's mastery.

Acquiring further professional knowledge has been found to increase nurses' sense of authority, autonomy and decision‐making power, and through sharing this knowledge, their motivation and love of the profession grow (Adib‐Hajbaghery et al. 2021; Bolandian‐Bafghi et al. 2022). This phenomenon resonated with the group as both our love of the profession was strengthened and our confidence in articulating the purpose and role of nursing's contribution to patient care and society was increased.

By examining our reflections, we can now consider that our collective intellectual nourishment, which gave us such joy, contributed significantly to our shared commitment to complete our studies.

#### 4.2.4 Professional Friendship and Socratic Inquiry

The group's professional friendship with each other aided the experience of professional growth and is closely aligned with but distinct from our insights relating to common humanity. It differs from this in that our love and joy flowed from the experience of learning as a professional group.

Joy, and the joy of learning really was when we were together. None of us talked about learning being joyful … the joy was in doing it together.

Our professional friendship was characterised by our mutual respect for each other as women, nurses and developing researchers, along with our interest and investment in each other's projects, constructive challenge and practical support. We later reflected on the evidence presented in the Communities of Practice (CoP) scoping review conducted by James‐McAlpine et al. (2023). The construct, purpose, motivations and benefits identified in the review strongly resonate with the group's reflections. The socially situated learning paradigms of a CoP are directly applicable to the context of our doctoral programme, as was the spontaneous and organic development of our WhatsApp group. There was no intention at the inception of the group to form a CoP; it was only retrospectively that we identified with the notion that we were a CoP, perhaps bringing into question whether future programmes can replicate our experience.

An important function of our professional friendship was how it contributed to our intellectual development. Through WhatsApp the group was highly responsive; it felt as if support was never more than an hour away, often less, meaning learning took place in real‐time. The accessibility and safety of this group also contributed to managing each other's distress. Given its impact, it is interesting that the use of the WhatsApp tool and the formation of the group was not a planned educational intervention deployed by the University Teaching Team; rather, it was natural and organic in its form and function.

Our discourse was frequently Socratic in nature, and we now recognise we were supporting the development of each other's ideas and critical thinking, an essential cognitive capability for nursing (Fink and Martyn‐Nemeth 2023). This cognitive process is necessary to make complex decisions, such as the development of new knowledge in the creation of original work through a professional doctorate, as explained by Bloom's Taxonomy of Learning (Dabney and Eid 2024). It is reasonable to argue that because of the nature of the group as experienced nurses and clinical leaders in our field, and in line with the Nursing and Midwifery Council's (NMC) four pillars of nursing practice (NMC 2015), we did engage, albeit unconsciously, in Socratic questioning. The non‐judgmental critique, encouragement and use of humour within the group enabled each member to share their vulnerability of not knowing and their search for knowingness at a time appropriate to them. As expected by the NMC (NMC 2015), this approach to developing others is also commensurate with the consultant level of practice defined by the Royal College of Nursing levels of nursing practice (RCN 2024).

The professional friendship of the group and the connection we felt between us allowed us the freedom to vent and rehearse leaving the group in a psychologically safe, (Edmondson and Lei 2014) caring space. The support we gave and received as a sisterhood was distinct from family and supervisor support, potentially enabling participants to experience manageable distress and preventing it from becoming unmanageable (Devos et al. 2017).

## 5 Conclusion

In this discussion paper, we explored the group experience of completing our university's first professional nursing doctorate programme. Through our individual and collective reflections, we developed insights into our experience and the meaning that we attributed to it. This paper does not claim to be informed by primary research conducted by the group, albeit that we drew on the principles of autoethnography for our method. We argue that the rigour of the meta‐reflection we undertook to explore why we stayed and what it meant to us has generated valuable insights for discussion for future and current doctoral students and university teaching teams. In examining the academic literature, many of our insights and experiences have subsequently been validated and therefore can potentially add to what is already known about successful doctoral completion. The most significant outcome, however, is that it has brought closure to the reflexive process for our group, whose experience of the doctoral programme has been formative beyond what we might have expected.

As the first programme cohort for the nursing doctorate in our area, we became a close and supportive group, and this was seen as contributing to our success. While this could be ascribed to our characteristics as doctoral students and the creation of a sisterhood, some acknowledgement must be given to the selection process. The love and joy felt by participants came from deepening our nursing knowledge. The common humanity and sisterhood we felt between each other gave the support that allowed us to discuss the possibility of leaving, whilst giving a group identity that secured our commitment to stay.

The group identity we developed was nourishing to us but was accompanied by family support; the group did not experience the absence of family support. Students should galvanise the support from family, friends and others who understand the doctoral journey and the ambition to succeed.

From this reflective account of our experience, we summarise our insights for others. We recommend that students select a research topic and question that excites them, thereby sustaining their motivation and commitment to their research participants and serving as a vehicle to explore nursing and contribute new knowledge to the profession. It could be argued that this can be augmented through some form of social interaction with others on the same self‐directed learning programme, the benefit of which may be reflective of a CoP. Future cohorts, supervisors and teaching teams could consider how the literature on CoP (James‐McAlpine et al. 2023) and academic persistence (Lew et al. 2020) might support academic development. Opportunities to enable organic relationship development could be systematically planned into programmes to facilitate CoP formation. However, it is also probable that generating a cohesive cohort group cannot be artificially driven by academic staff. For us, WhatsApp was instrumental in facilitating connectedness; we don't attribute the development of the group identity to it, but highly valued the access it gave us to each other, including the encouragement to find the physical and psychological space to vent and secure the time needed to write. We recommend that tutors encourage students to set up an online communication channel, such as WhatsApp or similar, at an early stage in their programmes.

The doctoral experience and attrition literature point to the solitary and lonely experience of doctoral study (Reilly and Fitzpatrick 2009; Morris et al. 2021); our example offers an alternate perspective. By creating time and space for social interaction within taught elements, and sharing this paper as a positive and encouraging example of what the doctoral journey could be like, tutors can increase the likelihood of students developing professional friendships and a collective cohort identity.

Finally, our process of reflection shared in this paper brought us a sense of reflexive closure we did not realise we needed or would benefit from. This closure has opened our minds to our post‐doctoral identities and horizons, allowing our sisterhood to disperse in a functional and harmonious way as we move onwards with our journeys. Based on this experience, we recommend that doctoral tutors and students attend to closure on completion of professional doctorates, beyond initial graduation celebrations.

## Author Contributions

Made substantial contributions to conception and design of the manuscript, acquisition of data and analysis and interpretation of data: H.A., S.B., K.L., E.S.‐H. Involved in drafting the manuscript and revising it critically for important intellectual content: H.A., S.B., K.L., E.S.‐H. Involved in reviewing and commenting on final draft: H.A., S.B., K.L., E.S.‐H. Given final approval of the version to be published. Each author should have participated sufficiently in the work to take public responsibility for appropriate portions of the content: H.A., S.B., K.L., E.S.‐H. Agreed to be accountable for all aspects of the work in ensuring that questions related to the accuracy or integrity of any part of the work are appropriately investigated and resolved: H.A., S.B., K.L., E.S.‐H.

## Ethics Statement

The authors have nothing to report.

## Consent

The authors have nothing to report.

## Conflicts of Interest

S.B. and K.L. are employed by Oxford Brookes University. H.A. holds an Associate Lecturer contract with Oxford Brookes University.

## Data Availability

The authors have nothing to report.
